# Pre-clinical remote undergraduate medical education during the COVID-19 pandemic: a survey study

**DOI:** 10.1186/s12909-020-02445-2

**Published:** 2021-01-06

**Authors:** Bita Shahrvini, Sally L. Baxter, Charles S. Coffey, Bridget V. MacDonald, Lina Lander

**Affiliations:** 1 School of Medicine, University of California at San Diego, La Jolla, California USA; 2 Viterbi Family Department of Ophthalmology and Shiley Eye Institute, University of California San Diego, La Jolla, CA USA; 3 Health Sciences Department of Biomedical Informatics, University of California San Diego, La Jolla, CA USA; 4 Department of Surgery, Division of Otolaryngology/ Head and Neck Surgery, University of California San Diego, La Jolla, California USA; 5 Department of Family Medicine and Public Health, University of California San Diego, La Jolla, CA USA; 6 Division of Medical Education, School of Medicine, University of California San Diego, 9500 Gilman Drive, MC-0606, La Jolla, CA 92093-0606 USA

**Keywords:** COVID-19, Medical education, Pre-clinical, Distance learning, Remote learning

## Abstract

**Background:**

The Coronavirus Disease 2019 (COVID-19) pandemic has necessitated a sudden transition to remote learning in medical schools. We aimed to assess perceptions of remote learning among pre-clinical medical students and subsequently to identify pros and cons of remote learning, as well as uncover gaps to address in ongoing curricular development.

**Methods:**

A survey was distributed to first- and second-year medical students at the University of California San Diego School of Medicine in March 2020. Frequencies of responses to structured multiple-choice questions were compared regarding impacts of remote learning on quality of instruction and ability to participate, value of various remote learning resources, living environment, and preparedness for subsequent stages of training. Responses to open-ended questions about strengths and weaknesses of the remote curriculum and overall reflections were coded for thematic content.

**Results:**

Of 268 students enrolled, 104 responded (53.7% of first-year students and 23.9% of second-year students). Overall, students felt that remote learning had negatively affected the quality of instruction and their ability to participate. Most (64.1%) preferred the flexibility of learning material at their own pace. Only 25.5% of respondents still felt connected to the medical school or classmates, and feelings of anxiety and isolation were noted negatives of remote learning. Most second-year students (56.7%) felt their preparation for the United States Medical Licensing Examination Step 1 exam was negatively affected, and 43.3% felt unprepared to begin clerkships. In narrative responses, most students appreciated the increased flexibility of remote learning, but they also identified several deficits that still need to be addressed, including digital fatigue, decreased ability to participate, and lack of clinical skills, laboratory, and hands-on learning.

**Conclusions:**

Videocasted lectures uploaded in advance, electronic health record and telehealth training for students, and training for teaching faculty to increase technological fluency may be considered to optimize remote learning curricula.

## Background

The Coronavirus Disease 2019 (COVID-19) pandemic has disrupted countless aspects of economy, society, and health. Medical schools have been challenged by the abrupt transition to remote learning, enacted to protect patients and students. With the unprecedented cancellation of in-person classes, small groups, and clinical experiences, this study aims to assess the relative successes and areas for improvement in a preclinical remote learning curriculum.

On March 16, 2020, the University of California San Diego (UCSD) School of Medicine (SOM) announced that all pre-clinical education would be conducted completely online and remotely. Prior to this announcement, the standard pre-clinical curriculum consisted of lecture-based organ system blocks, problem-based learning small groups, laboratory-based classes such as anatomy, histology, and ultrasound, and various pre-clinical electives. Clinical exposures included a course in doctoring/humanism (“Practice of Medicine”) and longitudinal ambulatory care apprenticeships, where students participated in weekly clinics with faculty mentors. Table 1 details these curricular components and changes associated with the remote learning transition. These changes were made abruptly in response to public health regulations and state-mandated orders [1] rather than being motivated by deliberate theoretical or conceptual pedagogical frameworks.

**Table 1:** Summary of UCSD School of Medicine pre-clinical curriculum before and after the transition to remote learning

Remote Learning Changes in the UCSD SOM Curriculum		
	Before the transition	After the transition
**Organ System Blocks**	2–4 h of consecutive lectures with videocasts made available following the in-person lectures. Final Exams taken via Examplify on personal computers on campus.	2–4 h of consecutive videocasted lectures, with all videocasts for the block uploaded in advance. Final Exams taken via Examplify on personal computers at students’ homes.
**Lab Classes (Anatomy, Histology, Ultrasound)**	In-person labs with lab manuals posted online in advance of scheduled lab times.	All labs cancelled. Optional, live videoconference office hours offered. Manuals posted online in advance of corresponding lab office hours.
**Practice of Medicine**	4-h small group sessions every other week. Groups of 8 students and 1 facilitator. In-person practice of physical exam/doctoring skills and student encounters with patient actors.	4-h videocasted small group sessions every other week. Groups of 8 students and 1 facilitator. No physical exam learning. Videoconference student encounters with patient actors.
**Problem Based Learning**	2-h in-person small group sessions twice a week to review patient cases and present relevant topics. Groups of 8 students and 1 facilitator.	2-h videoconference small group sessions twice a week to review patient cases and present relevant topics. Groups of 8 students and 1 facilitator.
**Ambulatory Care Apprenticeships (ACA)**	Students paired with a primary care preceptor in San Diego to practice conducting patient histories, physical exams, and writing notes.	Cancelled.
**Pre-clinical Electives**	Range from clinical preceptorships to community service to lecture based electives.	Some cancelled, others moved to videoconference platforms.

Remote learning has gained popularity in higher education over the last decade [2–8]. Medical schools have increasingly utilized videocasting and virtual learning platforms to afford greater flexibility for students [9–15]. However, integration of technology and flexible remote learning options into medical curricula has historically been relatively slow [16–20]. While students have had the option to view lectures online and purchase optional remote learning resources (e.g. question banks, video subscription services, and flashcards) [21], most preclinical knowledge was still disseminated in-person. Nonetheless, videocasted lectures and virtual learning platforms have impacted the way students engage with and participate in curricula, both of which are key aspects of health professions education according to the social learning perspective of situated learning theory [22]. The sudden and complete transition to remote learning necessitated by COVID-19 required rapid development of remote learning curricula to meet complex learning objectives. Rooted in the cognitive learning theory, we aimed to continue empowering learners with the necessary tools to progressively master fundamental knowledge in the pre-clinical curriculum [23].

The goals of this study were to better understand the effects of this complete transition to remote learning during the COVID-19 pandemic on pre-clinical students. To achieve this, we developed and deployed a survey of first- and second-year UCSD medical students. Because of the novelty and unprecedented nature of this transition, this was a hypothesis-generating study. The survey was motivated by an initial exploratory approach and entailed a wide array of survey items, including open-ended items, to evaluate student perspectives around this transition. By doing so, our goals were to better understand the relative successes and failures of the remote learning experience and to subsequently inform best practices for curriculum design, even after the COVID-19 pandemic resolves.

## Methods

### Study population

This study was conducted at the UCSD School of Medicine, an accredited allopathic medical school in La Jolla, CA. Eligible participants included all medical students enrolled in their first or second year as of March 30, 2020. The UCSD Institutional Review Board (IRB) approved this protocol as a quality improvement study. The average age for UCSD SOM’s entering class of 2019 is 24, with 58% of students being female, and the vast majority being California residents [24]. Students at UCSD engage in a traditional medical curriculum with the first year focused on normal physiology and basic sciences and the second year focused on anatomy, pharmacology, histology, and pathology. Training programs in clinical skills are woven throughout the pre-clinical curriculum longitudinally. The pre-clinical curriculum at UCSD SOM is entirely pass/fail with no internal rankings or influence on Alpha Omega Alpha (AOA) honors society selection, fostering a noncompetitive, tight-knit learning environment among students. Specific curricular components and changes associated with the remote learning transition are detailed in Table 1.

### Survey design and implementation

We developed the survey instrument (Appendix 1) based on prior annual student surveys. We consulted faculty, staff, and students to establish face validity, using a similar process as described in prior studies involving medical student surveys [25]. Students rated the value of various remote learning resources, aspects of curricular structure, communication from leadership, feelings of connectedness, out-of-pocket expenses, and suitability of their living environment. For second-year students, the survey also asked about preparedness for subsequent stages of training. Finally, open-ended questions asked students about telehealth experiences (e.g. healthcare delivery via remote technologies [26] such as virtual doctor-patient visits conducted over video), strengths and weaknesses of the remote curriculum, components that should be incorporated into the standard curriculum, and overall reflections.

We used an online software platform (Qualtrics, Provo, UT) to distribute the survey via e-mail to all eligible participants. Survey completion required approximately 10 min. The survey was anonymous, optional, and not linked to any student evaluations.

### Statistical analyses

Descriptive statistics were generated using the mean and standard deviations or counts/frequencies where appropriate, using Microsoft Excel Version 2004 (Microsoft Corporation, Redmond, WA, USA). To assess internal consistency reliability for structured/closed-ended survey items graded on the same Likert scale, Cronbach’s alpha calculations were performed using the *psych* package in R version 3.5.1 for each domain of the survey. Open-ended responses were coded by two independent coders (CC and BS) for thematic content. Comments were iteratively reviewed and mapped to various thematic domains. Discrepancies in emerging themes were reviewed by all co-authors until a consensus was reached. Representative comments demonstrating the major themes, chosen and agreed upon by all co-authors, were extracted for illustration. Sample size was based on a convenience sample of all pre-clinical students at our institution, aiming for rapid data acquisition regarding initial experiences of the remote learning curriculum soon after the transition. In addition, because we were not conducting a hypothesis-driven study, but rather an initial exploration of feedback to fuel future improvements in a rapidly changing environment, we did not perform formal power calculations, given that the sample size was logistically constrained by existing enrollments/class sizes.

### Ethical considerations

A key ethical consideration was assuring that students felt free to share honest feedback without any fear of repercussion. Two primary courses of action were taken in this regard. First, survey completion was entirely anonymous, without linkage of student names or identifying information to responses submitted. Second, the survey was optional, with no way to assess who had completed the survey and who had not. At the time of distribution, students were informed of both the anonymous and optional nature of the survey. We also informed students that there would be no possibility of linking their responses to their names and course evaluations. The pass/fail grading system further facilitated an environment for students to share direct, unfiltered feedback without fear of impacting their grades.

## Results

Of 268 students (134 in each class) invited to participate, 104 (38.8%) responded. Respondents consisted of 72 first-year students (53.7% response rate among all first-year students) and 32 second-year students (23.9%).

### Effects of remote learning on curricular components

For all curricular components besides lectures, most students felt the quality of instruction was somewhat or very negatively affected by the remote learning transition (Table 2). The Cronbach’s alpha for this domain of the survey was 0.89 (95% confidence interval 0.86 to 0.92). The highest proportions of students felt that remote learning had very negatively affected the quality of instruction in anatomy (49/74, 66.2%), ultrasound (39/47, 83.0%), and the ambulatory care preceptorship (51/53, 96.2%). Students felt that remote learning somewhat or very positively affected other curricular components, such as lecture-based learning (23/93, 24.73%) and problem-based learning (14/101, 13.9%). However, for the remaining curricular components, < 10% of students felt that the remote learning transition had any positive effects.

**Table 2:** Perceptions of the effects of remote learning on quality of instruction and on ability to participate among pre-clinical medical students at the University of California San Diego, March–April 2020. The number of respondents is indicated for each specific curricular component

	Very negatively affected	Somewhat negatively affected	Neutral	Somewhat positively affected	Very positively affected
**Effect of Remote Learning on Quality of Instruction**					
Lecture-based learning (*n* = 93)	8 (8.6%)	23 (24.7%)	39 (41.9%)	12 (12.9%)	11 (11.8%)
Problem-based learning (*n* = 101)	11 (10.9%)	41 (40.6%)	35 (34.7%)	10 (9.9%)	4 (4.0%)
Practice of Medicine (*n* = 77)	38 (49.4%)	32 (41.6%)	7 (9.1%)	0 (0.0%)	0 (0.0%)
Anatomy (*n* = 74)	49 (66.2%)	20 (27.0%)	4 (5.4%)	1 (1.4%)	0 (0.0%)
Histology (*n* = 74)	26 (35.1%)	27 (36.5%)	15 (20.3%)	4 5.41%	2 (2.7%)
Ultrasound (*n* = 47)	39 (83.0%)	7 (14.9%)	1 (2.1%)	0 (0.0%)	0 (0.0%)
Ambulatory Care Preceptorship (*n* = 53)	51 (96.2%)	0 (0.0%)	2 (3.8%)	0 (0.0%)	0 (0.0%)
Pre-clinical Electives (*n* = 69)	30 (43.5%)	17 (24.6%)	19 (27.5%)	3 4.35%	0 (0.0%)
**Effect of Remote Learning on Ability to Participate**					
Lecture-based learning (*n* = 95)	14 (14.7%)	17 (17.9%)	42 (44.2%)	11 (11.6%)	11 (11.6%)
Problem-based learning (*n* = 101)	8 (7.9%)	32 (31.7%)	46 (45.5%)	12 (11.9%)	3 (3.0%)
Practice of Medicine (*n* = 78)	17 (21.8%)	30 (38.5%)	26 (33.3%)	3 (3.9%)	2 (2.6%)
Anatomy (*n* = 73)	38 (52.1%)	20 (27.4%)	13 (17.8%)	1 (1.4%)	1 (1.4%)
Histology (n = 73)	33 (45.2%)	24 (32.9%)	14 (19.2%)	1 (1.4%)	1 (1.4%)
Ultrasound (*n* = 46)	37 (80.4%)	6 (13.0%)	2 (4.4%)	0 (0.0%)	1 (2.2%)
Ambulatory Care Preceptorship (*n* = 53)	45 (84.9%)	4 (7.6%)	3 (5.7%)	0 (0.0%)	1 (1.9%)
Pre-clinical Electives (*n* = 68)	25 (36.8%)	17 (25.0%)	22 (32.4%)	2 (2.9%)	2 (2.9%)

Perceptions of the effects of remote learning on ability to participate followed similar patterns. Besides lectures and problem-based learning, where students were neutral about their ability to participate remotely, the majority (> 60%) of students felt remote learning had somewhat or very negatively affected their ability to participate in all other curricular components (Table 2).

### Remote learning resources and curricular structure

**Fig. 1:**
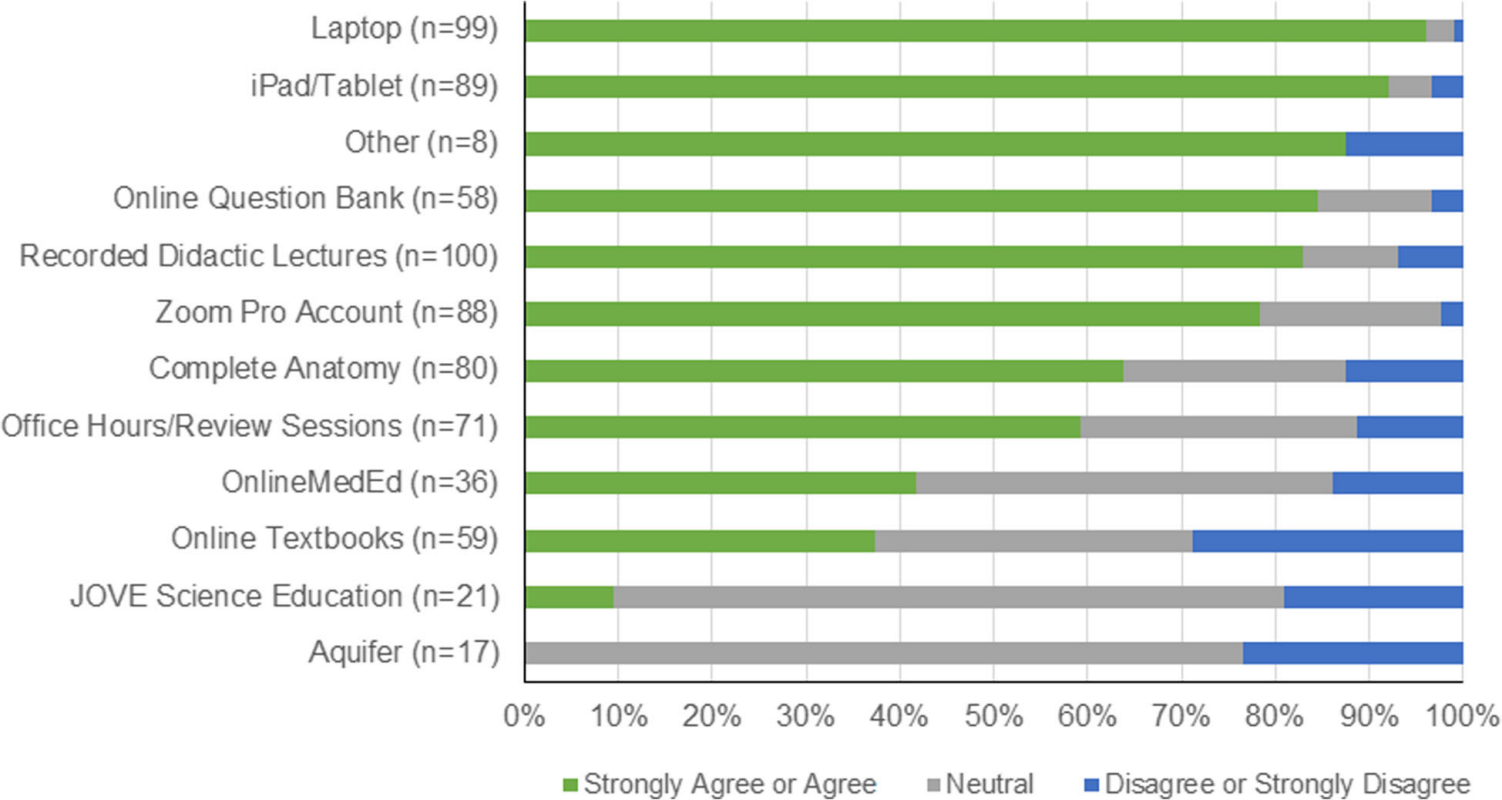
Perceptions regarding the value of various remote learning resources among pre-clinical (first- and second-year medical students) at the University of California San Diego, March–April 2020. Students were asked to rate their level of agreement with whether the specific remote learning resources were valuable for their medical education

Pre-clinical students endorsed variable utilization of remote learning resources (Fig. 1). The Cronbach’s alpha for responses regarding remote learning resources was 0.80 (95% confidence interval 0.75 to 0.86). Resources regarded as valuable by half or more of respondents included a laptop, tablet, online question bank subscription, recorded didactic lectures, videoconferencing software (Zoom, Zoom Video Communications, Inc., San Jose, CA, USA), digital anatomy education app (Complete Anatomy, 3D4Medical, Dublin, Ireland), and online office hours and review sessions (Fig. 1). Resources of relatively lesser value included online textbooks, Online MedEd (OnlineMedEd, Inc., Austin, TX, USA) and Aquifer (Aquifer, Lebanon, NH, USA) for clinically-oriented organ system and specialty-specific learning, and JoVE Science Education (JoVE, Cambridge, MA, USA) for physical exam skills learning. Almost two-thirds (66/103, 64.1%) of students preferred having the flexibility of learning material at their own pace rather than having required modules and set due dates. When asked about the ideal frequency of due dates, most (55/103, 53.4%) preferred weekly due dates. Fewer students preferred due dates to occur daily (4/103, 3.9%), every few days (15/103, 14.6%), biweekly (16/103, 15.5%), or monthly (13/103, 12.6%).

### Costs, living arrangements, and connectedness

For most (72/103, 69.9%) students, transitioning to remote learning incurred less than $100 of additional out-of-pocket expenses. However, almost a quarter (24/103, 23.3%) spent $101–$500, and there were 7 students (6.8%) who spent over $500 during the remote learning transition.

With remote learning, one-fifth of the students (20/98, 20.4%) moved outside the greater metropolitan area surrounding the institution. The remaining students stayed locally, whether in their current housing arrangements (72/98, 73.5%) or moving to different housing nearby (6/98, 6.1%). About one-fifth of students (21/98, 21.4%) felt their living arrangements were not conducive to remote learning (Table 3). This was primarily attributed to lack of quiet study space, a barrier identified by a quarter of students (24/98, 24.5%). Very few students (5/98, 5.1%) indicated lack of sufficient internet or technology. The responses regarding home learning environments had a Cronbach’s alpha of 0.76 (95% confidence interval 0.68 to 0.83).

**Table 3:** Living arrangements and feelings of connectedness among pre-clinical (first- and second-year medical students) at the University of California San Diego, March–April 2020

Statements (*N* = 98 students)	Scale of Agreement				
	Strongly Disagree	Disagree	Neutral	Agree	Strongly Agree
Overall, my current living arrangements are conducive to remote learning.	6 (6.1%)	15 (15.3%)	17 (17.4%)	40 (40.8%)	20 (20.4%)
I have access to sufficient internet to meet the demands of remote learning.	1 (1.0%)	8 (8.2%)	7 (7.1%)	40 (40.8%)	42 (42.9%)
I have access to sufficient technology (i.e. a computer with a webcam, iPad, etc) to meet the demands of remote learning.	0 (0%)	5 (5.1%)	2 (2.0%)	43 (43.9%)	48 (50.0%)
Given my living arrangements, I have sufficient access to quiet study space to meet the demands of remote learning.	7 (7.1%)	17 (17.4%)	16 (16.3%)	38 (38.8%)	20 (20.4%)
Given the transition to remote learning, I still feel connected to UCSD School of Medicine.	14 (14.2%)	38 (38.8%)	21 (21.4%)	20 (20.4%)	5 (5.1%)
Given the transitions to remote learning, I still feel connected to my classmates.	20 (20.4%)	31 (31.6%)	20 (20.4%)	26 (26.5%)	1 (1.0%)

Overall, students felt less connected during remote learning. Only about a quarter of students still felt connected to the medical school or to their classmates (Table 3).

### Preparation for subsequent stages of training

Second-year students felt that remote learning negatively affected their preparation for subsequent stages of training. Over half (17/30, 56.7%) felt that their preparation for the United States Medical Licensing Examination (USMLE) Step 1 examination was negatively affected. About a quarter (7/30, 23.3%) felt their preparation was positively affected, and the remainder felt their preparation had not changed. Similarly, 13 (43.3%) felt unprepared for clinical clerkships, 9 (30.0%) felt prepared, and the remainder were neutral.

### Narrative results

Four dominant themes emerged from 254 unique narrative responses to open-ended questions:

I. *Structure – flexibility and efficiency*

Many students praised the increased flexibility afforded by remote learning. Almost two-thirds (38/59, 64.4%) cited increased flexibility as the best part of remote learning (Table 4), noting the benefits of self-pacing, which permitted them to pause and work out difficult concepts, or to speed up recordings to enhance efficiency. Many appreciated the opportunity to “get ahead” of the lecture schedule via pre-recorded lectures (Table 5a, b). Several students noted they were able to coordinate studies with their circadian rhythms to optimize periods of productivity and efficiency (Table 5c, d). Students also valued the flexibility to engage in activities such as research, Step 1 studying, self-care, and volunteering (Table 5e). Several students praised remote learning for the time and financial gains from eliminating commutes, either from home to school or from one building on campus to another (Table 5f).

**Table 4:** Free responses among pre-clinical (first- and second-year medical students) at the University of California San Diego, March–April 2020. Best and worst components of the remote learning curriculum and aspects that should be continued in future, hybrid curricula

What are the best components of the remote curriculum? (*N* = 59)	N (%)	Which components of the remote curriculum should be continued in the standard curriculum in the future? (*N* = 54)	N (%)	What gaps remain in the remote curriculum? (*N* = 61)	N (%)
Increased flexibility	38 (64%)	Videocasted lectures, uploaded in advance for the block	27 (50%)	Clinical skills learning (POM, ACA)	31 (51%)
Videocasted lectures, uploaded in advance for the block	18 (31%)	Virtual PBL	9 (17%)	Lab classes (Anatomy, Histology, Ultrasound)	15 (25%)
Increased efficiency	10 (17%)	Increased office hours/review sessions	7 (13%)	Zoom/Digital Fatigue	11 (18%)
Increased office hours/review sessions	4 (7%)	Extra resources/practice questions	6 (11%)	Difficult to participate/ask questions	8 (13%)
Virtual PBL	4 (7%)			Disorganization/ Lacking structured schedule	7 (11%)
				Poor communication/ Transparency	5 (8%)

**Table 5:** Student quotations representative of views expressed by multiple students regarding thematic content of free response questions

**I. Structure: Flexibility and Efficiency**	
**a.** “I do not learn well in the physical lecture session bc I cannot pause and address confusion right away.”	
**b.** “I appreciate having all the block’s lectures at the beginning so I can watch them at my own pace and get ahead if I choose.”	
**c.** “I am an early riser so I do like being able to wake up early and start on lectures by 6 AM.”	
**d.** “The ability to learn more or less depending on the energy level of the day is absolutely massive. And has been the STRONGEST addition to my education, regardless of the circumstance.”	
**e.** “More freedom to productively use my time (research/STEP studying).”	
**f.** “I also commute to school so remote learning is saving me a lot of money and time, which I am very happy about.”	
**g.** “The biggest killer in remote learning is time self management... I sleep in more than I should and do less work than I should. I would like help keeping myself accountable by having more assignments with more set due dates. If the assignments are there, I will do them.”	
**h.** “At grad housing, my neighbors above have children who are persistently loud and while at home my large family are all working and always on calls. Many students feel that it’s difficult to focus, be engaged, and be placed in an environment conducive to learning. As such, a student like myself who used to never struggle with having motivation to get to work is having more difficulties now than ever to simply be a student.”	
**II. Remote Learning Format: Digital Fatigue and Participation**	
**i.** “Zoom classes feel much longer online than in person.”	
**j.** “POM [Practice of Medicine] for 4 h straight is impossible. Many of the activities are difficult to do over Zoom. Honestly, the past few weeks after logging off the 4 h POM zoom call, I have been so discouraged that I couldn’t focus for the rest of the evening. Four hours on a Zoom call is physically and emotionally draining.”	
**k.** “Interaction with faculty and students is simply not the same. It’s a bit hard to describe, but as someone who is very much an “in person” and “tangible” learner, going to lecture and seeing the faculty, speaking with peers, and interacting with everyone in person is more conducive to my learning style.”	
**l.** “It’s very hard to do any sort of group studying. It’s very hard to find a quiet place to study. It’s very hard to learn doctoring skills … There is no way to practice your skills/ask questions.”	
**III. Content: Lab Classes and Clinical Skills Learning**	
**m.** “As someone who learns best through hands on methods, I’m struggling with anatomy and POM and really missing the opportunity to practice patient interviewing/physical exams during ACA. I do not feel prepared for an OSCE at the end of the year.”	
**n.** “As of now I do not feel prepared for step style questions, or comfortable trying to apply my knowledge in relevant clinical settings.”	
**o.** “It has also been hard not having clinical experiences, since that was my favorite part of medical school.”	
**p.** “Anatomy is extremely difficult to learn remotely - and I know a lot of students who have just resigned to not learning pelvic anatomy given the circumstances.”	
**q.** “(Telehealth participation) has been very valuable and a great learning experience and is helping me stay grounded and connected to my role as a med student.”	
**r**. “Telehealth... Was a great learning experience, practiced taking a hx, presenting to attending, writing a note.”	
**s.** “(Telehealth participation) has been hugely helpful for my motivation and keeping up with my interviewing skills.”	
**IV. Mental Health: Anxiety and Isolation**	
**t.** “Being more or less alone for the past ~ 2 months has showed me the importance of connection and social interaction in my own mental well-being and the role that our in-person classes served in meeting that need for me. I miss in-person class for that reason the most. And I would be worried that if the SOM switched to more remote learning permanently after COVID-19, a lot of student’s mental health would decline due to isolation and lack of relationships with classmates. “	
**u.** “It’s an incredibly isolating experience... mental health is more so a challenge than ever with all of this, and it is impacting all facets of our student life: academic performance, extracurricular commitments, socializing, etc. “	
**v.** “Some of us are being hit more by the complete psychological lack of interaction, that can’t really be remedied by looking at boxes on a computer with friends and mentors faces in them.”	
**w.** “In addition to family issues and regular coursework, it’s more difficult to go about daily activities, such as grocery shopping or exercising; some of us do not have access to a quiet study space with reliable internet; some of us are managing free clinic responsibilities, where more administrative duties are falling on students. Some of us have had a known exposure to COVID-19 ourselves or have responsibilities to our communities outside of school. “	
**x.** “I also wish I knew what was going on—I get so many emails from the school and UC San Diego Health that I don’t know what to open for actual information about my own curriculum, etc.”	

By contrast, some students struggled with decreased structure, citing that it was easy to fall behind (Table 5g). When asked specifically about gaps in the remote learning curriculum, 7/61 respondents (11.5%) noted lack of a structured schedule and disorganization (Table 4). Others conveyed that productivity and motivation were hindered by home environments that were not conducive to studying (Table 5h).

II. *Remote learning format – digital fatigue and participation*

Prolonged engagement in remote learning formats proved problematic for many students. Numerous respondents (11/61, 18.0%) specifically reported digital fatigue as a significant drawback of the remote curriculum. Interestingly, some students noted the greatest digital fatigue with synchronous small group sessions, which were designed to be interactive, but instead led to disengagement, exhaustion, and inability to focus (Table 5i, j). Some respondents felt remote formats hampered participation due to technical issues and inability to study in groups or effectively use office hours (Table 5k, l). In contrast, some students felt the remote format enhanced small group interactions, and 9/54 respondents (16.7%) desired that virtual problem-based learning (PBL) be continued beyond the pandemic period (Table 4).

III. *Content gaps – lab classes and clinical skills learning*

Unsurprisingly, when asked specifically about the biggest gaps in the remote learning curriculum, 31/61 respondents (50.8%) cited clinical skills learning (Table 4). Many students noted deficiencies with history-taking and physical exam training. The overall dearth of clinical skills training left students feeling unprepared for clinical assessments and encounters (Table 5m, n). Students also felt a loss of motivation when clinical training opportunities disappeared (Table 5o). Most students also felt that digital substitutes were inadequate for lab classes like anatomy, histology, and ultrasound (Table 5p).

However, a handful of students who participated in telehealth encounters found the experience quite valuable. Fourteen students reported participating in some form of telehealth since transitioning to remote learning, the majority (78.6%) of whom obtained the experience via a student-run Free Clinic for uninsured members of the San Diego community. While students had mixed feelings regarding the effectiveness and efficiency of telehealth patient visits, students valued opportunities to participate in patient care and derived motivation from these visits (Table 5q-s).

IV. *Mental health – anxiety and isolation*

In the standard curriculum, pre-clinical students typically learned together in-person, thus developing close-knit relationships with each other. The transition to remote learning and the accompanying isolation took a noticeable toll on students’ mental health (Table 5t-v). A notable proportion of students (11/66 respondents, 16.7%) mentioned isolation, feelings of disconnectedness, or declining mental health in their responses. Anxiety and uncertainty made it difficult for many to focus on academics. Disruption of normal routines and additional stresses from stay-at-home orders made remote learning particularly trying for some students (Table 5w). An incessant barrage of mixed and sometimes contradictory information was also difficult to navigate, and several students noted that effective communication from medical school administration was necessary to assuage feelings of uncertainty and maintain a positive educational environment (Table 5x).

## Discussion

The COVID-19 pandemic forced medical schools around the world to transition their pre-clinical curricula to remote learning platforms overnight [27]. Unsurprisingly, this posed immense challenges for administrators, course directors, and students alike [28–31]. Recent articles have discussed the impact of COVID-19 on medical education [27, 32–34], but these have largely consisted of editorial or opinion pieces without data demonstrating students’ perspectives. Few studies have captured the experience of pre-clinical medical students [30, 31, 35–38], and several that did, have focused on a single curricular component such as anatomy, offering only a narrow glimpse into students’ experience [30, 31].

Because circumstances did not permit sufficient time to re-design the entire pre-clinical curriculum tailored specifically to remote learning, our institution migrated the majority of the structure and content of the existing curriculum to videoconference formats, with the exception of several components which were cancelled outright. Our survey results suggest that pre-clinical students had mixed feelings about this approach, finding some aspects of this remote learning curriculum beneficial and others detrimental both to their studies and mental health. Our key findings were that (1) pre-clinical students felt the loss of clinical experiences acutely, (2) the learning experiences in laboratory-based classes were particularly negatively impacted, and (3) students enjoyed the increased flexibility afforded by remote learning, particularly as it pertained to videocasted lectures.

### Clinical skills learning: shortfalls and opportunities for growth

Given that clinical experiences constituted a small portion of the pre-clinical curriculum, it was surprising to learn the profound effect of losing these experiences on pre-clinical students’ motivation and morale. Students explained that direct patient care experiences fueled their motivation to keep up with the academic rigors of medical school. This observation highlights the value of increasing clinical exposure during the first 2 years of medical school, as many institutions have done [39–43]. The lack of sufficient clinical skills practice also appears to be a commonly noted limitation among medical students surveyed at other institutions [35, 36].

Our study revealed that bolstering medical student participation in telehealth may be a potential solution to address this challenge. Others have proposed implementation of virtual, group-based interprofessional education (IPE) to discuss and solve clinical vignettes as a way to bridge the inevitable gap in clinical reasoning skills [38]. Prior studies have also found that interaction with telehealth during medical school contributes to improved core competencies, medical knowledge, overall learning and higher quality patient care [44]. The increased reliance on telemedicine should motivate allocation of structured time in the pre-clinical curriculum for telehealth training, which may include electronic health record (EHR) training as well, to equip students with the practical skills they will need to succeed in an evolving clinical landscape.

### Negative impacts on lab class experiences

Our cohort of first-year respondents offered unique insight into student perspectives regarding digitally simulated anatomy compared to traditional cadaveric dissection and prosection. Most students were not satisfied with learning anatomy remotely, and several commented that online platforms were not adequate replacements for in-person learning with anatomic specimens. However, our results may have been affected by the fact that the remote learning anatomy curriculum at our institution was passive; lab manuals were posted online, and instructors hosted optional, live office hours to answer questions. Remote anatomy instruction with more active student engagement and directed activities may be more successful.

With several medical schools moving away from cadaveric dissections and towards online platforms such as Complete Anatomy, 3D printed organs, and virtual reality to teach anatomy [45–49], our survey results contribute to the discussion about whether or not these methods enhance pre-clinical anatomy learning from the student perspective. Moreover, as others have noted, unique ethical issues come into play when trying to integrate human donor dissections with videoconferencing tools used for remote learning [30]. Moving forward, medical educators may consider a hybrid approach to optimize the pre-clinical anatomy curriculum by combining traditional laboratory dissection with remote learning resources to augment learning wherever possible.

### Increased flexibility offered a positive experience

Similar to results of other survey studies [36, 37], the ability to engage in self-paced learning due to schedule flexibility and early availability of pre-recorded lectures was highly valued by UCSD students in this survey. Nearly two-thirds of students praised increased flexibility as the single best aspect of the remote learning curriculum. Additionally, with optimized efficiency afforded by increasing the speed of videocasted lectures and the ability to work ahead, students found more time to engage in extracurricular activities like research, Step 1 preparation, and self-care.

While there was generally positive sentiment regarding the opportunity for increased efficiency, students noted that the abruptness of the transition brought some challenges. Technical difficulties with videoconferencing posed an initial challenge for many faculty members, which detracted from some students’ learning. Efforts to improve digital “fluency” by training faculty in the fundamentals of remote teaching technologies should help to ensure a more consistent and successful experience. Digital fatigue was also frequently cited as a barrier to student engagement and efficient learning. Future initiatives to design more effective remote learning curricula might mitigate digital fatigue by replacing small group sessions lasting 3 or 4 h with multiple, shorter modules.

There is growing debate about whether in-person lectures for pre-clinical medical education are necessary, and whether medical schools should pursue centralized online content as the primary basis for didactic teaching [9, 50–54]**.** Opting for the latter could create opportunities for multi-institutional teaching consortia and shared learning platforms, potentially freeing the resources of medical educators at home institutions to focus on more individualized instruction and clinical experiences.

### Limitations

This study’s design as a single-center analysis limits broader generalizability to other settings or institutions. Additionally, we focused solely on medical students’ experiences, which may not apply to other health sciences students such as pharmacy or nursing students. About half of pre-clinical students responded to the survey; there may have been response bias leading to overrepresentation of those with the strongest feelings, either positive or negative. To preserve the anonymity of the students surveyed, we did not ask about demographic factors and therefore could not analyze students’ perceptions of remote learning by age, gender, or race/ethnicity. This limits our ability to understand the potentially differential effects of these curricular changes in different student populations, particularly those from traditionally under-represented groups. This is an important limitation, as prior frameworks have shown that under-represented students may have different needs and priorities for their medical education [55]. Finally, this survey represented a method of soliciting early feedback from students shortly after an abrupt curricular transition. With more time and stability, and the easing of government-imposed restrictions, future curricular changes will be more firmly guided by conceptual frameworks, and hypothesis-driven analyses and/or interventions will be an important area of ongoing investigation. Moreover, remote learning has quickly evolved in the time since this study was completed. As a result, current student perceptions and experiences with remote learning may vary given that students may now be more used to learning remotely, and medical schools have had additional time to develop more robust remote learning curricula.

## Conclusions

Remote learning had some negative impacts on pre-clinical learning, chiefly related to the loss of clinical experiences in the core curriculum, reduced impact of laboratory courses, and heightened feelings of anxiety and isolation. However, students also perceived positive aspects of remote learning including increased flexibility, opportunities to explore different learning resources, and time to focus on wellness. Given the likelihood that periodic disruptions to medical education due to new or resurgent pandemics will continue, it is imperative that medical schools develop sustainable remote learning curricula. This includes implementing structured EHR and telehealth training time within the core curricula for students and developing fluency in remote teaching formats and technologies amongst medical educators. The COVID-19 pandemic has created opportunities to expand the role of remote learning in medical education, and this study provides valuable insight for educators participating in re-designing preclinical curricula to effectively meet the needs of students.

## Data Availability

The datasets used and analysed during the current study are available from the corresponding author on reasonable request.

## Appendix

### Qualtrics survey instrument

**Pre-Clinical Remote Learning Quality Improvement.**

Q1 What year are you currently enrolled in at the school of medicine?

o MS1 (1).

o MS2 (2).

Q2 How has the transition to remote learning affected the **quality of instruction** you receive for each of the following curricular components?

**Table:**

	Very negatively affected (1)	Somewhat negatively affected (2)	Neutral (3)	Somewhat positively affected (4)	Very positively affected (5)	N/A: I did not take this course via remote learning (7)
Lecture-based learning (1)	o	o	o	o	o	o
PBL (2)	o	o	o	o	o	o
POM (3)	o	o	o	o	o	o
Anatomy (4)	o	o	o	o	o	o
Histology (6)	o	o	o	o	o	o
Ultrasound (7)	o	o	o	o	o	o
ACA Preceptorship (8)	o	o	o	o	o	o
Pre-clinical electives (9)	o	o	o	o	o	o

Q3 How has the transition to remote learning affected **your participation** in each of the following curricular components?

**Table:**

	Very negatively affected (1)	Somewhat negatively affected (2)	Neutral (3)	Somewhat positively affected (4)	Very positively affected (5)	N/A: I did not take this course via remote learning (6)
Lecture-based learning (1)	o	o	o	o	o	o
PBL (2)	o	o	o	o	o	o
POM (3)	o	o	o	o	o	o
Anatomy (4)	o	o	o	o	o	o
Histology (5)	o	o	o	o	o	o
Ultrasound (6)	o	o	o	o	o	o
ACA Preceptorship (7)	o	o	o	o	o	o
Pre-clinical electives (8)	o	o	o	o	o	o

Q4 I find the following resources valuable for my remote learning.

**Table:**

	Strongly Disagree (1)	Disagree (2)	Neutral (3)	Agree (4)	Strongly Agree (5)	N/A: I have not used this resource (6)
Online question bank subscription (1)	o	o	o	o	o	o
Online textbooks (2)	o	o	o	o	o	o
Podcasted lectures (4)	o	o	o	o	o	o
Online office hours/review sessions (5)	o	o	o	o	o	o
Zoom Pro Account (6)	o	o	o	o	o	o
Complete Anatomy (7)	o	o	o	o	o	o
JOVE Science Education (8)	o	o	o	o	o	o
Aquifer (9)	o	o	o	o	o	o
OnlineMedEd (10)	o	o	o	o	o	o
iPad/Tablet (11)	o	o	o	o	o	o
Laptop (12)	o	o	o	o	o	o
Other: (13)	o	o	o	o	o	o

Q5 Since implementation of remote learning, how much additional money have you paid out of pocket for educational resources?

o $0–100 (5).

o $101–$500 (2).

o $501–1000 (3).

o $1000+ (4).

Q6 I felt adequately prepared to transition to remote learning.

o Strongly Disagree (1).

o Disagree (2).

o Neutral (3).

o Agree (4).

o Strongly Agree (5).

Q7 In terms of structure for the remote learning curriculum, which do you prefer?

o I prefer the flexibility of learning material at my own time and pace (1).

o I prefer having required modules and due dates (2).

o No preference (3).

Q8 Hypothetically, if some learning modules were required to be completed by certain due dates, how frequently would you prefer those due dates to occur?

o Daily (1).

o Every few days (2).

o Weekly (3).

o Biweekly (4).

o Monthly (5).

Q9 I have found the following methods of communication effective in informing me about the changes in my education.

**Table:**

	Strongly Disagree (1)	Disagree (2)	Neutral (3)	Agree (4)	Strongly Agree (5)	N/A: I have not received communication via this method (6)
Emails (1)	o	o	o	o	o	o
Canvas (2)	o	o	o	o	o	o
Virtual Town Halls with SOM Deans (3)	o	o	o	o	o	o
Facebook Class Page (4)	o	o	o	o	o	o
University Website (5)	o	o	o	o	o	o
Other: (6)	o	o	o	o	o	o

Q10 Given the transition to remote learning, I feel that the examination process provides a fair assessment.

o Strongly Disagree (1).

o Disagree (2).

o Neutral (3).

o Agree (4).

o Strongly Agree (5).

Q11 How do you feel your USMLE STEP 1 preparation has been affected by the transition to remote learning for your medical education curriculum?

o Very negatively affected (1).

o Somewhat negatively affected (2).

o No change (3).

o Somewhat positively affected (4).

o Very positively affected (5).

Q12 Given the transition to remote learning, how prepared do you feel to begin clerkships?

o Very unprepared (1).

o Somewhat unprepared (2).

o Neutral (3).

o Somewhat prepared (4).

o Very prepared (5).

Q13 Since implementation of remote learning, which of the following describes your residence location:

o Stayed at UCSD graduate student housing (1).

o Stayed in existing off-campus housing in San Diego (5).

o Moved off-campus in San Diego (2).

o Moved outside San Diego area (4).

Q14 Rate your agreement with the following statements:

**Table:**

	Strongly Disagree (1)	Disagree (2)	Neutral (3)	Agree (4)	Strongly Agree (5)
I have access to sufficient internet to meet the demands of remote learning. (2)	o	o	o	o	o
I have access to sufficient technology (ie a computer with a webcam, iPad, etc) to meet the demands of remote learning. (3)	o	o	o	o	o
Given my living arrangements, I have sufficient access to quiet study space to meet the demands of remote learning. (4)	o	o	o	o	o
Overall, my current living arrangements are conducive to remote learning. (6)	o	o	o	o	o

Q15 Rate your agreement with the following statements:

**Table:**

	Strongly Disagree (1)	Disagree (2)	Neutral (3)	Agree (4)	Strongly Agree (5)
Given the transition to remote learning, I still feel connected to UCSD School of Medicine. (1)	o	o	o	o	o
Given the transition to remote learning, I still feel connected to my classmates. (2)	o	o	o	o	o

Q16 If you have participated in any form of tele-health during this time, please comment on your experience:

Q17 What are the best components of the remote learning curriculum?

Q18 What gaps remain in the remote learning curriculum?

Q19 Which components of the remote learning curriculum should be continued in the standard curriculum in the future?

Q20 Finally and most importantly- please reflect on your own experience with remote learning for the pre-clinical curriculum. What would you like for course directors or others in medical education to better understand about the experience?

## Abbreviations

COVID-19: Coronavirus Disease 2019
UCSD: University of California San Diego
IRB: Institutional Review Board
